# A Substitute for Rubber Gloves

**Published:** 1904-07

**Authors:** 


### A SUBSTITUTE FOR RUBBER GLOVES.

Murphy is now experimenting with a temporary covering for the hands while operating. He immerses his hands in a four to eight per cent. solution of gutta percha in benzine. This leaves the hands covered by an impervious and sufficiently durable rubber coating. This film is very thin and does not interfere with the delicacy of touch. The false skin can readily be removed by washing the hands in benzine.

